# The Effects of Acid on Calcium and Phosphate Metabolism

**DOI:** 10.3390/ijms25042081

**Published:** 2024-02-08

**Authors:** Juan D. Salcedo-Betancourt, Orson W. Moe

**Affiliations:** 1 Department of Internal Medicine, Division of Nephrology, University of Texas Southwestern Medical Center, Dallas, TX 75390, USA; 2 Charles and Jane Pak Center for Mineral Metabolism and Clinical Research, University of Texas Southwestern Medical Center, Dallas, TX 75390, USA; 3 Department of Physiology, University of Texas Southwestern Medical Center, Dallas, TX 75390, USA

**Keywords:** phosphate, calcium, acidosis

## Abstract

A variety of changes in mineral metabolism aiming to restore acid–base balance occur in acid loading and metabolic acidosis. Phosphate plays a key role in defense against metabolic acidosis, both as an intracellular and extracellular buffer, as well as in the renal excretion of excess acid in the form of urinary titratable acid. The skeleton acts as an extracellular buffer in states of metabolic acidosis, as the bone matrix demineralizes, leading to bone apatite dissolution and the release of phosphate, calcium, carbonate, and citrate into the circulation. The renal handling of calcium, phosphate and citrate is also affected, with resultant hypercalciuria, hyperphosphaturia and hypocitraturia.

## 1. Introduction

**Figure 1:**
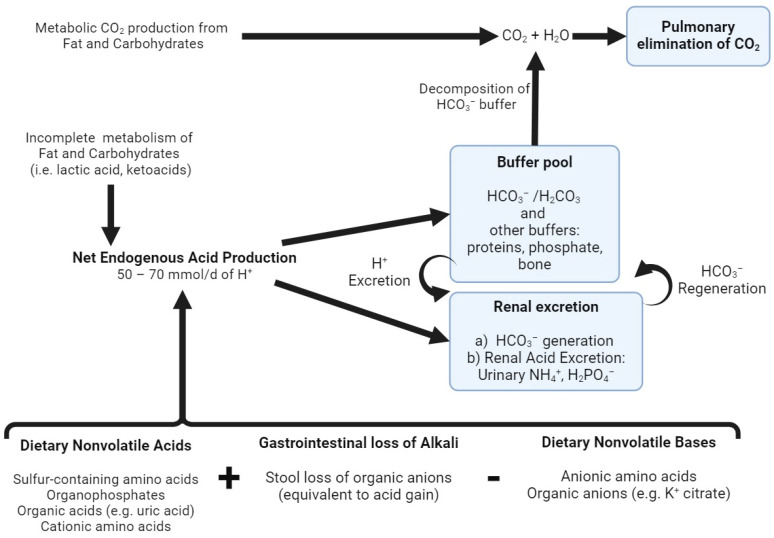
Acid–base homeostasis. The complete oxidation of fat, carbohydrates and neutral amino acids generates CO_2_, which is eliminated via ventilation by the lungs. Net endogenous acid production (NEAP) comprises endogenous acids produced from the incomplete metabolism of fat and carbohydrates (e.g., lactic acid, ketoacids), dietary nonvolatile (fixed) acids and the stool loss of organic anions (equivalent to acid gain) after subtracting the dietary sources of nonvolatile bases (e.g., potassium citrate). This H^+^ production is continuously buffered by various buffer systems and is ultimately excreted by the kidneys in the form of urinary ammonium (NH_4_^+^) and titratable acids (i.e., H_2_PO_4_^−^). Additionally, the kidneys are responsible for the reclamation of filtered HCO_3_^−^, as well as HCO_3_^−^ generation, to replenish the decomposed buffer pool. Created with BioRender.com, accessed on 23 December 2023.

Several mechanisms contribute to the buffering and elimination of excessive H^+^ to maintain acid–base balance in response to acid loading. As a first-line defense, acids are buffered by intra- and extracellular buffers, followed by elimination by the kidneys and the lungs (Figure 1). Carbon dioxide (CO_2_) is a volatile acid generated from the metabolic production or decomposition of bicarbonate buffer and is eliminated through ventilation. Maintaining a constant CO_2_ concentration set by respiratory control regulates arterial pH in conjunction with the bicarbonate buffer system (H^+^ + HCO_3_^−^ ⇌ H_2_CO_3_ ⇌ CO_2_ + H_2_O). Intracellular and extracellular non-HCO_2_ buffers include phosphates, hemoglobin and a variety of proteins, (NH_2_-protein-COO^−^ + H^+^ ⇌ NH_2_-protein-COOH + H^+^ ⇌ NH_4_^+^-protein-COOH) [1]. Additionally, the inorganic component of bone can buffer a significant amount of H^+^ during acid loading.

Both calcium and phosphate metabolism are involved in acid–base homeostasis at several physiological intersections. Phosphate plays a key role in defense against metabolic acidosis, both as an intracellular and extracellular buffer, as well as in the renal excretion of excess H^+^ in the form of urinary titratable acid through this buffering reaction (Na_2_HPO_4_ ⇌ HPO_4_^2−^+ 2Na and HPO_4_^2−^ + H^+^ ⇌ H_2_PO_4_^−^) [2]. The skeleton acts as an extracellular buffer in states of metabolic acidosis, as the acid-induced dissolution of bone hydroxyapatite releases Ca^2+^ and phosphate into the extracellular fluid (ECF) [3].

The kidneys participate in the reabsorption of filtered and the regeneration of decomposed bicarbonate, which is tantamount to the excretion of fixed (nonvolatile) acids. Renal net acid excretion (NAE = V·[(U_NH4_^+^ + U_TA_) − (U_HCO3_^−^ + U_Cit_^2−/3−^)], where V = volume of urine excreted per unit time, UNH_4+_ = urinary ammonium concentration, UTA = urinary titratable acid concentration, UHCO_3_^−^ = urinary bicarbonate concentration and UCit^2−/3−^ = Urinary citrate concentration. In response to metabolic acidosis, there is a net increase in renal NAE, due to an increase in both UNH_4+_ and UTA and a decrease in UCit^2−/3−^ excretion and urine pH) refers to the elimination of renal acid required to balance fixed (nonvolatile) acid production. Ammonia (NH_3_) is synthetized in the proximal tubule (PT) from the metabolism of amino acids. NH_3_ binds H^+^ ions and is excreted into the urine as NH_4_^+^ (NH_3_ + H^+^ ⇌ NH_4_^+^). The oxidation of glutamine generates two molar equivalents of NH_4_^+^ and two of HCO_3_^−^; as a result, an equivalent amount of HCO_3_^−^ is returned to the body when NH_4_^+^ is excreted in the urine [4]. Titratable acids refer to several weak acids that carry H^+^ (e.g., phosphate, creatinine, uric acid, citrate and ketoanions) [5], account for about one third of renal acid excretion (NAE) and are quantified collectively by titrating urine back to a blood pH of 7.4 (hence, titratable acid). An important titratable acid is monovalent phosphate (H_2_PO_4_^−^), due to its favorable pKa of 6.8. Creatinine, on the other hand, with a pKa of 4.9, has a more significant buffer capacity when urinary pH falls below 6 [2].

## 2. Acid–Base Homeostasis and Gastrointestinal Calcium–Phosphate Handling

In normal physiology, the gastrointestinal system plays a crucial role in maintaining acid–base balance through gut fluid and electrolyte transport. The stomach secretes HCl (hydrochloric acid), which is balanced by the simultaneous release of bicarbonate ions by the pancreas into the small intestine (70–120 mmol/L). The small amount of alkali lost in the stool in the form of organic anions (approximately 30 mmol/day) is balanced by daily renal net acid excretion (Figure 1). However, conditions that disrupt normal gastrointestinal function can lead to disturbances in acid–base balance. Vomiting and nasogastric drainage can result in metabolic alkalosis, a process initiated by H^+^ loss and sustained by disproportionate chloride loss compared to other electrolytes, leading to increased serum bicarbonate and hypokalemia. Secretory diarrhea (and high-output ileostomy drainage) can cause significant stool organic anion and volume losses surpassing the kidney’s ability to maintain acid–base balance, leading to hyperchloremic metabolic acidosis and hypokalemia. Similarly, biliary and pancreatic drainage can lead to metabolic acidosis due to the loss of a bicarbonate-rich fluid when the drainage volume exceeds 2 L/day. Understanding these mechanisms and potential disturbances is crucial for managing acid–base imbalances in gastrointestinal diseases [6].

### 2.1. Normal Gastrointestinal Calcium and Phosphate Handling

**Figure 2:**
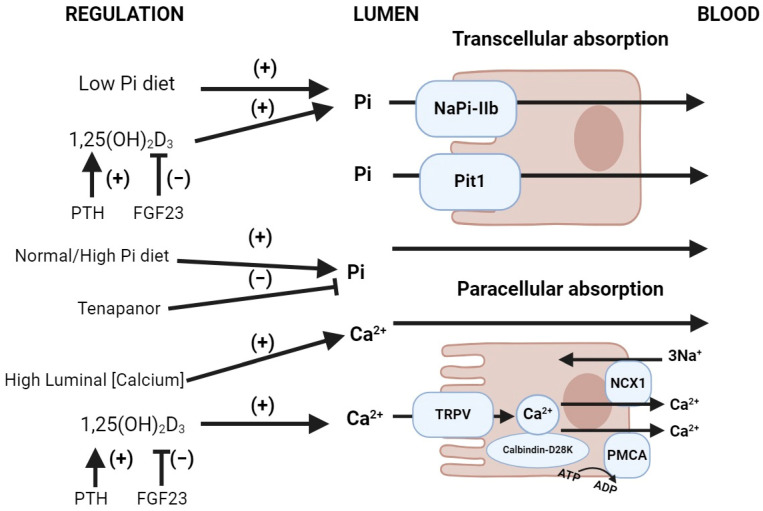
Normal gastrointestinal handling of phosphate and calcium. Upper image: Intestinal phosphate absorption. NaPi-IIb (Slc34A2) is upregulated by calcitriol (1,25(OH)_2_D_3_), downregulated by the FGF-23-mediated inhibition of 1α-hydroxylase (which converts 25(OH)D_3_ into 1,25(OH)_2_D_3_) and the activation of 24-hydroxylase (which converts 25(OH)D_3_ into its inactive metabolite 24,25(OH)_2_D_3_). A normal/high-phosphate diet increases the luminal phosphate gradient, favoring paracellular phosphate reabsorption. A low-phosphate diet favors transcellular phosphate reabsorption due to the upregulation of NaPi-IIb as well as the reduction in the luminal phosphate gradient. Tenapanor, a sodium/hydrogen exchanger-3 (NHE3) inhibitor, blocks paracellular phosphate reabsorption. The sodium-dependent transporter Pit1 (Slc20A1) has a minor role in intestinal phosphate uptake compared to NaPi-IIb. Lower image: Intestinal calcium absorption. Calcitriol (1,25(OH)_2_D_3_) regulates active calcium intestinal absorption by upregulating the brush border membrane calcium transporter TRPV6. Additionally, calcitriol increases the enterocyte synthesis of calbindin-D28K, favoring the absorption of calcium into the microvilli. Abbreviations: Pi = inorganic phosphate, PMCA = plasma membrane calcium ATPase. Created with BioRender.com, accessed on 23 December 2023.

The average dietary intake of calcium is 500 to 1000 mg (12.5 to 25 mmol)/day. Calcium absorption occurs in the intestine through paracellular pathways as well as calcitriol-dependent transcellular calcium transporters (predominantly TRPV6 and, to a lesser extent, TRPV5) [7]. Additionally, calcitriol increases the intestinal epithelial cell synthesis of calbindin-D28K, allowing calcium to bind calbindin-D28K and unloading the calcium–calmodulin complex, favoring the absorption of calcium (Figure 2) [8,9].

A normal adult ingests 30–45 mmol of phosphorus or 900 to 1400 mg phosphorus equivalent (although there is no elemental phosphorus in the diet)/day. Dietary phosphate is found both in inorganic (orthophosphate and pyrophosphate acid) and organic forms (phosphoprotein, phospholipids, phosphoglycans, nucleic acids). While meat tends to have both monovalent (H_2_PO_4_^−^) and divalent phosphate (HPO_4_^2−^), fruits with a pH below 6.8 have more phosphate in the monovalent form (H_2_PO_4_^−^) [2]. There is low-to-zero bioavailability of plant-based dietary phosphate primarily in the form of phytate, which is only minimally absorbed due to the lack of the enzyme phytase in the human small intestine [10]. The small intestine has a significantly higher phosphate absorptive capacity compared to the colon [11], which becomes relevant during conditions of extremely high luminal phosphate concentrations, such as during the use of phosphate enemas [12].

Intestinal phosphate absorption occurs through paracellular transport via passive diffusion through tight junction complexes driven by electrochemical gradients across epithelia (Figure 2), and active transcellular transport through sodium-dependent phosphate co-transporters. It is conjectured that paracellular phosphate permeability is influenced by intracellular pH [13]. Tenapanor is a phosphate absorption inhibitor that targets the paracellular pathway via enteric apical membrane sodium/proton exchanger-3 inhibition [14]. NaPi-IIb (Slc34A2) is a Type II sodium–phosphate co-transporter present at the enterocyte brush border membrane (BBM) and is responsible for the majority of sodium-dependent transcellular intestinal phosphate uptake [15]. NaPi-IIb is upregulated by 1,25(OH)_2_-dihydroxyvitamin D_3_ (1,25(OH)_2_D_3_) and as well as low dietary phosphate [16]. The 1,25(OH)_2_D_3_ regulation of intestinal phosphate absorption depends on transcription involving the vitamin D receptor (VDR), whereas low dietary phosphate enhances sodium-dependent phosphate transport through post-transcriptional mechanisms independently of 1,25(OH)_2_D_3_ [17,18]. Additionally, FGF-23 inhibits 1α-hydroxylase which converts 25(OH)D_3_ to 1,25(OH)_2_D_3_ and activates 24-hydroxylase (the elimination pathway of 25(OH)D_3_), leading to decreased 1,25(OH)_2_D_3_ levels and the downregulation of NaPi-IIb (Figure 2) [19]. The type III sodium-dependent Slc20 transporters PiT1 (Slc20A1) and PiT2 (Slc20A2) also participate in phosphate absorption [16]. Both PiT1 and PiT2 are ubiquitously expressed and proposed to act as “housekeeping” transporters in the basolateral membrane of enterocytes. Additionally, PiT1 is expressed in the apical membrane of enterocytes [20], likely playing a minor role in intestinal phosphate uptake compared to NaPi-IIb [21,22].

### 2.2. Gastrointestinal Calcium Handling in Metabolic Acidosis

There are conflicting data regarding the influence of metabolic acidosis in intestinal calcium absorption. Some studies suggest that metabolic acidosis impairs intestinal calcium reabsorption via the suppression of 1α-hydroxylase [23] and decreased concentrations of 1,25(OH)_2_D_3_ [24]. Other studies, however, have not found suppression of 1,25(OH)_2_D^3^ production in normal adults [25], nor a reduction in the intestinal absorption of calcium or phosphorus after the induction of metabolic acidosis [26]. There are reports of either increased [27,28] or decreased [29] intestinal calcium absorption after alkali administration.

### 2.3. Gastrointestinal Phosphate Handling in Metabolic Acidosis

Historically, there have been conflicting data regarding the role of metabolic acidosis in intestinal phosphate absorption [26,30,31]. Recently, Stauber et al. showed an increase in intestinal phosphate absorption upon the induction of metabolic acidosis in a mouse model, with the stimulation of sodium-dependent phosphate uptake occurring due to an increase in the expression of the NaPi-IIb cotransporter in the BBM of the small intestine [32]. From a physiologic viewpoint, acidosis increases phosphate release from the bone and phosphaturia, so increased gut absorption is a fitting compensation.

## 3. Acid–Base Homeostasis and Renal Calcium–Phosphate Handling

### 3.1. Normal Renal Calcium and Phosphate Handling

**Figure 3:**
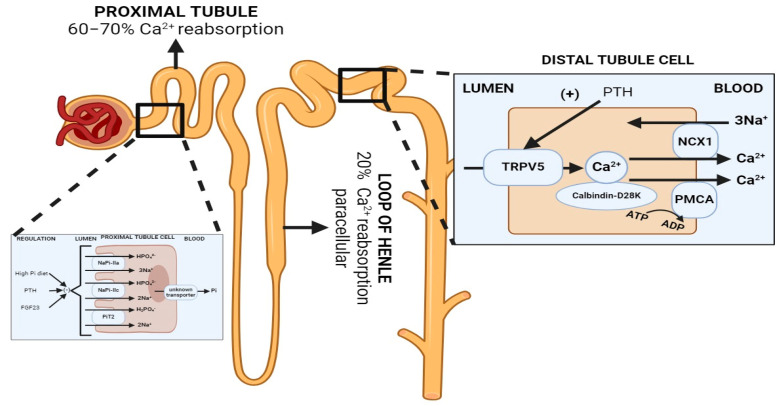
Normal renal handling of phosphate and calcium. Left: Phosphate handling. Phosphate is predominantly reabsorbed in the proximal tubule. PTH and FGF-23 promote the retrieval and degradation of NaPi-IIa (Slc34A1), NaPi-IIc (Slc34A3) and PiT2 (Slc20A2). Calcitriol is believed to increase phosphate reabsorption in the proximal tubule, but the effects are confounded since it also increases PTH levels. Right: Calcium handling. 60–70% of filtered calcium is passively reabsorbed with sodium and water in the proximal tubule, and 20% in the thick ascending limb via paracellular mechanisms, and 10–20% is reabsorbed transcellularly through TRPV5, an apical membrane calcium channel in the distal tubule and collecting duct. PTH enhances distal calcium reabsorption through the upregulation of TRPV5, leading to increased serum calcium levels. Abbreviations: Pi = inorganic phosphate, NCX = Na^+^/Ca^2+^ exchanger, PMCA = plasma membrane calcium ATPase. Created with BioRender.com, accessed on 23 December 2023.

Total body calcium stores are approximately 1000 g (25,000 mmol), mostly in the form of hydroxyapatite in the mineralized bone matrix (99%) [33]. Calcium homeostasis is balanced through three organ systems: intestinal, bone and kidney. In the kidney, 60–70% of filtered calcium is passively reabsorbed with sodium and water in the proximal tubule, and 20% in the thick ascending limb, both via paracellular mechanisms, and the remaining 10–20% is reabsorbed transcellularly through the apical calcium channel TRPV5 and extruded through the basolateral Na-Ca exchanger and Ca-ATPase, in the distal tubule and collecting duct (Figure 3). The regulatory mechanisms of renal calcium reabsorption adapt to the body’s needs, with reabsorption increased by hypocalcemia, PTH release and volume contraction [34]. Plasma ionized calcium is the main regulator of PTH secretion, where in the setting of hypercalcemia, CaSR is stimulated, which inhibits PTH secretion on parathyroid chief cells. PTH secretion enhances bone turnover through osteoblast activation and stimulates the 1α-hydroxylase conversion of 25(OH)D_3_ into 1,25(OH)_2_D_3_, which, in turn, increases the intestinal absorption of calcium. Additionally, PTH production is inhibited by 1,25(OH)_2_D_3_ [35]. At the tubular level, PTH enhances distal calcium reabsorption through the upregulation of TRPV5, leading to increased serum calcium levels [36].

Phosphate is essential for many physiological needs, including cellular energy production, cell signaling and structure, oxygen delivery to tissues and bone mineralization, among others. Most phosphate exists as hydroxyapatite in the mineralized bone matrix (85%), and 14% intracellularly [34], of which 70% occurs as organic (phospholipids, phosphoproteins, nucleic acids, adenosine triphosphate (ATP) and cyclic adenosine monophosphate (cAMP)) and 30% as inorganic phosphate. Only 1% of total body phosphate exists as non-osseous extracellular serum phosphate [36] in the serum, of which 15% is found to be protein bound, 14% is complexed to cations [35], and 47% is ionized as divalent (HPO_4_^2−^) and monovalent (H_2_PO_4_^−^) phosphate in a 4:1 ratio at pH 7.4 [36].

Phosphate homeostasis is balanced through three major systems: intestinal uptake, bone release/incorporation and renal excretion. Systemic phosphate balance is mainly regulated through changes in the urinary fractional excretion of phosphate under typical conditions [37,38]. Urinary phosphate contributes to 50% of titratable acids. Hence, renal phosphate excretion plays a significant role in renal net acid excretion (NAE) [39]. Only the ingestion of divalent phosphate (HPO_4_^2−^) and the urinary excretion of monovalent phosphate (H_2_PO_4_^−^) represents a net loss of H^+^ and a net gain of HCO_3_^−^; in contrast, the ingestion and excretion of monovalent phosphate (H_2_PO_4_^−^), does not represent either a gain or loss of base [40].

Phosphate is freely filtered by the glomerulus (about 90% of plasma phosphorus). Then, 80–90% of renal phosphate is reabsorbed in the proximal tubule. Reabsorption is sodium-dependent as it is performed by the sodium–phosphate co-transporters NaPi-IIa (Slc34A1) and NaPi-IIc (Slc34A3) located in the proximal tubule apical membrane [41]. Hence, renal phosphate excretion is determined by the glomerular filtration rate and the tubular maximum reabsorption rate (TMP-phosphate). Phosphaturia occurs when maximal tubular phosphate reabsorption becomes saturated. The regulatory mechanisms of renal phosphate reabsorption adapt to the body’s needs, [36] including the parathyroid hormone (PTH), Fibroblast Growth Factor-23 (FGF-23) and αKlotho [42]. FGF-23 and PTH promote the retrieval and degradation of NaPi-IIa [43], NaPi-Iic [41,44] and PiT2 (Figure 3) [8]. Additionally, in a phosphate depletion state, there is a reduction in the phosphaturic effect of PTH [36] due to the augmentation of the tubular reabsorption of phosphate [45]. It has been recently described that the calcium-sensing receptor (CaSR) represents a phosphate-sensing mechanism in the parathyroid gland, since raising the extracellular phosphate concentration significantly inhibited CaSR activity via non-competitive antagonism and stimulated PTH secretion in murine models [46].

### 3.2. Renal Calcium Handling in Metabolic Acidosis

**Figure 4:**
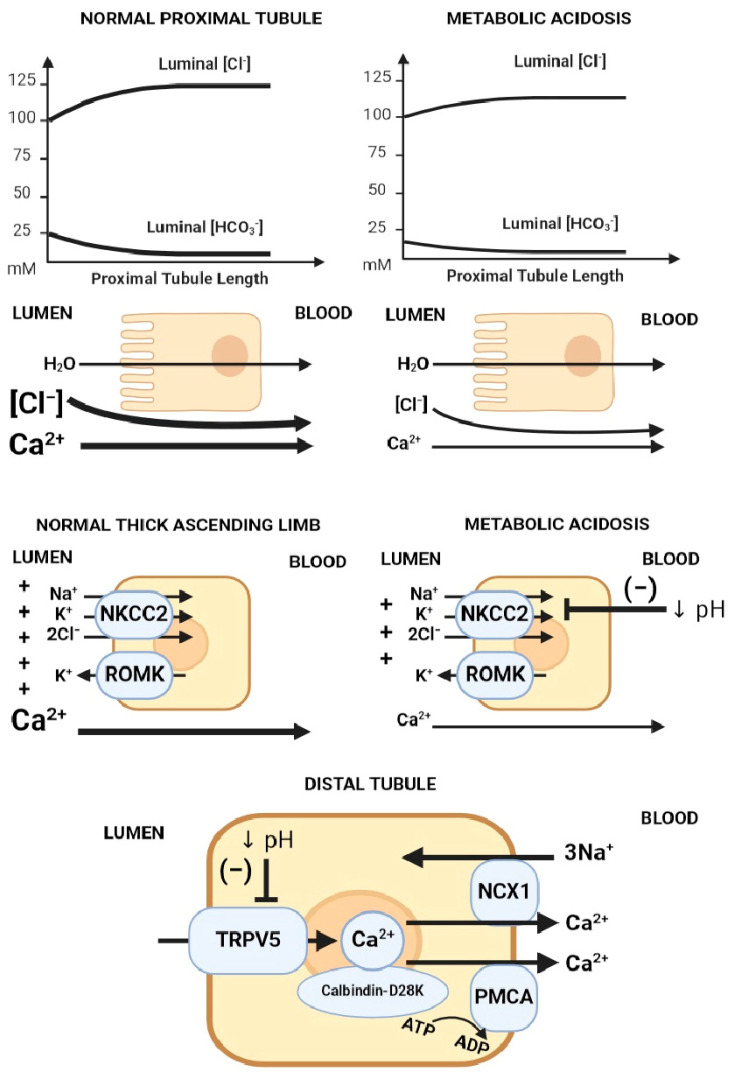
Mechanisms of acidosis-induced hypercalciuria. Proposed mechanisms of hypercalciuria in metabolic acidosis axially down the nephron: (1) Decreased electrochemical gradient required for paracellular calcium reabsorption in the proximal tubule. Luminal calcium is concentrated through the isotonic absorption of NaCl and water. Removal of HCO_3_^−^ from the lumen increases luminal [Cl^−^], creating an electrochemical gradient for paracellular calcium reabsorption. In metabolic acidosis, there is lower luminal HCO_3_^−^, leading to a blunted rise in luminal [Cl^−^], which translates into a lower chemical driving force for calcium reabsorption. (2) The suppression of Na/K/2Cl transport decreases the electrical gradient required for paracellular calcium reabsorption in the thick ascending limb (TAL). (3) TRPV5 gating inhibition caused by acidification of either extracellular or intracellular pH. Abbreviations: ↓ pH = Decreased pH. Created with BioRender.com, accessed on 23 December 2023.

Metabolic acidosis increases urinary calcium excretion, leading to negative calcium balance when persistent [26]. There are several proposed mechanisms to explain the hypercalciuric effect of metabolic acidosis (Figure 4) [47]: (1) the decreased electrochemical gradient required for paracellular calcium reabsorption in the proximal tubule, (2) the decreased driving force and permeability for paracellular calcium reabsorption in the thick ascending limb (TAL) via the claudin 16/19 complex [48], and (3) the direct inhibitory effect on TRPV5 gating caused by the acidification of either extracellular or intracellular pH.

Calcium reabsorption by the proximal tubule is carried primarily via the paracellular route, driven electrochemically by the luminal calcium concentration due to isotonic water and solute (mainly sodium chloride) absorption; hence, the main regulator of calcium flux in the proximal tubule is sodium chloride/water reabsorption. In metabolic acidosis, there is a blunted rise in luminal chloride compared to when serum bicarbonate is normal, which translates into a lower driving force for calcium absorption. In the thick ascending limb, calcium is absorbed primarily via the paracellular route, driven by positive luminal voltage (generated via sodium reabsorption via NKCC2 (Na-K-2Cl cotransporter) and potassium recycling via ROMK). Metabolic acidosis inhibits apical NaCl absorption via NKCC2, leading to reduced luminal voltage and decreased paracellular calcium reabsorption at the TAL segment. On the other hand, calcium reabsorption in the distal tubule mainly occurs via the transcellular route through the TRPV5 calcium apical calcium channel, after which calcium binds calbindin-D28K in the cytosol, which facilitates diffusion through the cytosol and eventual extrusion across sodium/calcium exchangers (NCX1) and calcium/ATPase (PMCA) on the basolateral membrane (Figure 4).

### 3.3. Renal Phosphate Handling in Metabolic Acidosis

Urinary phosphate excretion is predominantly determined by PTH and FGF-23. However, disturbances in acid–base balance also influence renal phosphate handling. During metabolic acidosis, there is a significant increase in the renal excretion of phosphate [5,49], which contributes to acid removal through titratable acid. As kidney function declines in chronic kidney disease (CKD), there is a decrease in ammonium formation and secretion by the proximal tubule [50]. There is an increase in the excretion of monovalent phosphate (H_2_PO_4_^−^) as a compensation through metabolic acidosis-induced phosphaturia [49]. Lower serum bicarbonate is associated with higher renal phosphate excretion in patients with CKD [51].

**Figure 5:**
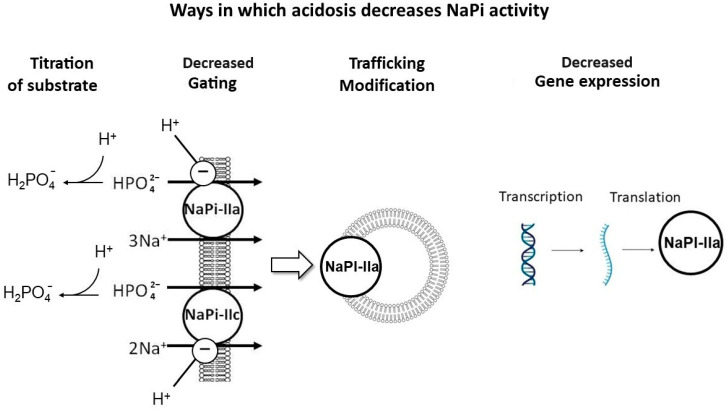
Mechanisms of acidosis-induced phosphaturia. Proposed mechanisms to explain the phosphaturic effect of metabolic acidosis: (1) Titration of substrate with H^+^, decreasing the luminal availability of divalent phosphate (HPO_4_^2−^), the preferred substrate to be absorbed. (2) Direct inhibitory gating of H^+^ on NaPi-IIa and IIc. (3) NaPi-IIa trafficking modifications, including impaired delivery of NaPi-IIa to the apical brush border membrane (BBM) and/or enhanced internalization from the BBM. (4) Impaired transcription and translation of NaPi-IIa. Created with BioRender.com, accessed on 23 December 2023.

There are several mechanisms proposed for this effect (Figure 5): (1) titration of the substrate with H^+^, decreasing the luminal availability of divalent phosphate (HPO_4_^2−^) for uptake; (2) the direct inhibitory gating of H^+^ on NaPi-IIa and NaPi-IIc [41]; (3) NaPi-IIa trafficking modifications, including the impaired delivery of NaPi-IIa to the apical BBM and/or enhanced internalization from the BBM [52]; and (4) the reduced transcription and translation of NaPi-IIa [49].

Metabolic acidosis has been shown to induce the expression of the phosphaturic hormones FGF-23 [53] and PTH [54,55,56,57]. Chronic metabolic acidosis for over 12 h led to the impaired transcription and translation of NaPi-IIa and decreased BBM Na–Pi cotransport activity. However, after the induction of acute metabolic acidosis (less than 6 h of acid loading), there was decreased Na-Pi cotransport activity, independent of changes in Slc34A1 mRNA expression [49]. The decrease in the apical NaPi-IIa protein in acute metabolic acidosis may be mediated by either the impaired delivery of NaPi-IIa to the BBM or enhanced internalization from the apical BBM [49,52]. Others have attributed acid-induced phosphaturia to the direct effects of local pH on the flux of these co-transporters in the proximal tubule [41], which was further confirmed with transcriptome analysis of acid-loaded murine kidneys that revealed a decrease in both NaPi-IIa and NaPi-IIc mRNA abundance, while the abundance of Pit1 and Pit2 transporters remained unchanged despite the induction of metabolic acidosis [41]. Nowik et al. showed that NaPi-IIa KO mice (*Slc34A1*−/−) had no acid-induced phosphaturia, while WT mice had a progressive reduction in BBM Na/Pi cotransport activity during ammonium chloride-induced metabolic acidosis, suggesting that metabolic acidosis-induced phosphaturia is caused by an inhibitory effect of low pH on the flux of NaPi-IIa [41].

Besides metabolic acidosis, increased phosphaturia is also seen with acute respiratory acidosis [58]. Severe phosphate depletion, on the other hand, tends to result in hyperchloremic metabolic acidosis due to the impaired proximal capacity for bicarbonate reclamation [59].

## 4. Acid Effect on Calcium–Phosphate in Bone

**Figure 6:**
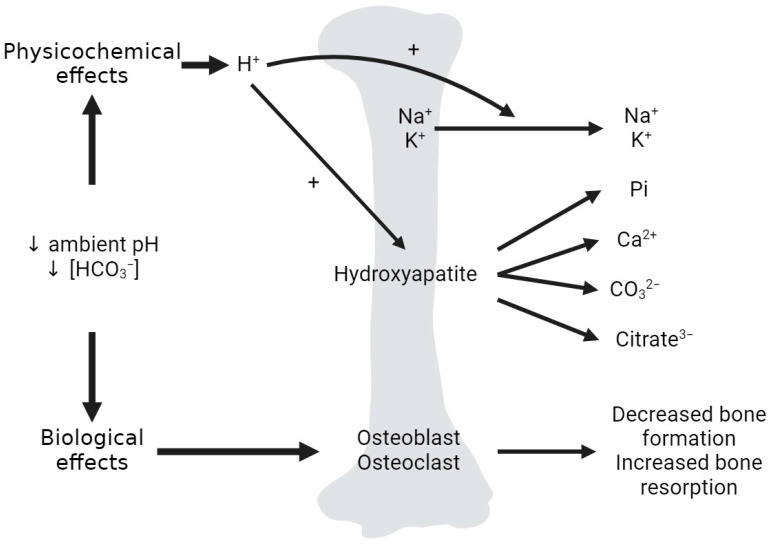
Effects of metabolic acidosis on the bone. Dual effects of acid on bone. The physicochemical effects of acid on the bone include the exchange of incoming H^+^ for Na^+^ and K^+^ at the bone matrix surface, as well as the dissolution of hydroxyapatite, releasing phosphate (Pi), calcium, carbonate (CO_3_^2−^) and citrate (citrate^3−^) from the bone matrix into the circulation. The biological effects include low ambient pH and [HCO_3_^−^] effects on osteoblasts, osteoclasts and osteocytes, leading to a net decrease in osteoblastic bone formation and an increase in osteoclastic bone resorption. Created with BioRender.com accessed on 23 December 2023.

Most of the total body calcium and phosphate stores exist as hydroxyapatite (Ca_10_(PO_4_)_6_(OH)_2_) in the mineralized bone matrix (99%) [33,60]. Although the quantitative significance of bone in acid buffering has been questioned by some [61], it is widely accepted that in states of metabolic acidosis, there is buffering of H^+^ by the bone, preserving systemic pH. Acid loads induce the bone release of sodium, potassium, phosphate, calcium, carbonate, and citrate (Figure 6) [62,63]. Of note, the bone mineral dissolution and resorption, as well as the additional buffering of H^+^ by the bone effects, are far less pronounced in respiratory acidosis [64]. With chronic metabolic acidosis, there is a decrease in osteoblastic bone formation, an increase in osteoclastic bone resorption and a decrease which can result in osteomalacia and osteoporosis [3].

### 4.1. Bone, Acid and Calcium Homeostasis

Upon the induction of metabolic acidosis, there is a rapid, albeit small, increase in serum calcium concentration and urine calcium excretion, with no change in intestinal calcium absorption [28,65]. When acid is infused in nephrectomized rodents, there is a rapid increase in serum calcium, representing dissolution of the bone mineral phase [66]. It is likely that the mineral phase of the bone is the source for the increase in urinary calcium excretion in metabolic acidosis [67]. Metabolic acidosis induces the physicochemical dissolution of bone hydroxyapatite, which releases phosphate and calcium salts (calcium carbonate) into the extracellular fluid (ECF) (Figure 6) [68], with subsequent calciuria [69]. Micropuncture studies found that acidosis decreases renal calcium reabsorption, which is rectified with the correction of acidosis [70]. The administration of alkali therapy with sodium bicarbonate in patients with distal renal tubular acidosis (RTA) and positive acid balance reduces urinary calcium excretion [71]. Increased rates of acid production that are unmatched by increased renal net acid excretion (NAE) lead to bone buffering and hypercalciuria, which increases the risk of calcium-containing kidney stones [72] and osteoporosis [73]. However, patients with isolated familial proximal RTA exhibit normal urinary calcium excretion related to their calcium intake, indicating that chronic metabolic acidosis alone may be insufficient to disturb calcium balance [74]. Hence, it is conceivable that positive acid balance, acidemia, and increased urinary calcium excretion are necessary for the development of metabolic acidosis-induced bone complications [68].

### 4.2. Bone, Acid and Phosphate Homeostasis

The total bone content of phosphate is about 20,000 mmol. The bone continuously exchanges phosphate with the extracellular space, at approximately 100 mmol per day, with a positive bone phosphate balance during growth (zero in normal adults, and negative in the elderly) [4]. The release of phosphate from bone during metabolic acidosis has been documented in both in vivo and in vitro models [75,76,77], as has a slight increase in circulating phosphate during metabolic acidosis, an effect attributed to the acid-stimulated release of phosphate from bone along with calcium and carbonate (Figure 6) [68]. Additionally, phosphate depletion has been shown to increase bone resorption, resulting in an alkali load discharge into the extracellular fluid [59]. Some propose that phosphate retention could protect against the bone resorption effects of acidosis by independently inhibiting bone resorption [78].

### 4.3. Bone, Acid and Citrate Metabolism

The bone matrix harbors over 90% of total body citrate [79], which is derived from differentiated osteoblasts and associates and strengthens hydroxyapatite crystals [80]. Citrate plays a key role in acid–base balance as a potential buffer when released from the bone in metabolic acidosis, but since it is metabolizable, citrate is also an important base-equivalent. When bone is exposed to acidic pH and undergoes physicochemical dissolution, phosphate, calcium, carbonate, and citrate are released from the bone matrix into the circulation (Figure 6) [81]. Additionally, metabolic acidosis potentially increases osteoblast citrate production. Chronic metabolic acidosis leads to increased proximal tubule citrate reabsorption, leading to base-conserving hypocitraturia. In combination with acidosis-induced hypercalciuria, this results in an increased risk of nephrolithiasis [82].

## 5. Conclusions

A variety of changes in mineral metabolism aiming to restore acid–base balance occur in acid loading and metabolic acidosis. The integrated physiological responses to acid include the sequestration of H^+^ by various extracellular and intracellular buffers, increased CO_2_ elimination through ventilation, augmented renal HCO_3_^−^ and citrate reabsorption, and acid elimination through the excretion of H^+^ carried by ammonia and buffers, including phosphate. Additionally, the bone matrix demineralizes, leading to bone apatite dissolution and the release of phosphate, calcium, carbonate, and citrate into the circulation. The renal handling of calcium, phosphate and citrate is also affected, with resultant hypercalciuria, hyperphosphaturia and hypocitraturia. While the primary purposes of these adaptations are to act as evolutionary conserved defenses against acid loading, the “trade-off” to the system is the occurrence of some undesirable compromises, such as bone and stone complications. The distinction between “state” and “disease” depends on the nature, severity and duration of the acid load and the capacity of the defense. The understanding of the pathophysiology of the mineral complications of acidosis will equip us with the ability to prevent and ameliorate these conditions.
